# Can hospital accreditation enhance patient experience? Longitudinal evidence from a Hong Kong hospital patient experience survey

**DOI:** 10.1186/s12913-019-4452-z

**Published:** 2019-09-03

**Authors:** Ellie Bostwick Andres, Wen Song, Wei Song, Janice Mary Johnston

**Affiliations:** Univeristy of Hong Kong, School of Public Health, Patrick Manson Building, (North Wing), 7 Sassoon Road, Pokfulam, Hong Kong

**Keywords:** External quality assessment, Patient satisfaction, Hospital medicine, Accreditation

## Abstract

**Background:**

Hospital accreditation is expected to improve health care quality and patient satisfaction. However, little and conflicting evidence is currently available to support its effect on patient outcomes, particularly patient experience. Hong Kong recently launched a pilot programme to test an infrastructure for accreditation of both private and public hospitals with the Australian Council on Healthcare Standards. This study aims to evaluate the longitudinal impact of hospital accreditation on patient experience in a publicly-funded university teaching hospital in Hong Kong.

**Methods:**

Three cross-sectional surveys were conducted at three time points: 9 months pre- accreditation as baseline (T1), three (T2) and fifteen months (T3) post-accreditation. Acute care inpatients aged 18 to 80 were recruited on the second day of hospital admission to complete the Picker Patient Experience Questionnaire-15 (PPE-15). Baseline data was first compared to the 2005 Hong Kong average for public hospitals using t-tests. Data was then analyzed using ANOVA and multiple linear regression to evaluate differences across the three cross-sections and examine the effect of accreditation over time while controlling for covariates.

**Results:**

3083 patients (T1 = 896, T2 = 1093, T3 = 1094) completed the survey for a response rate of 83.5, 86.1, and 83.8%, respectively. The hospital baseline domain and summary patient experience scores differed from the Hong Kong public hospital average obtained from the 2005 Thematic Household Survey. All domain and summary patient experience scores declined (improved) over the study period (T1 to T3). The multiple regression results confirmed the time point score comparisons with declining (improving) parameter estimates for T2 and T3 for all domain and summary scores except the ‘continuity and transition’ domain, for which the declining coefficient was only significant at T3.

**Conclusions:**

While hospital accreditation has not been shown to improve patient outcomes, this study suggests the accreditation exercise may enhance patient experience. Moreover, it suggests the quality improvement initiatives associated with accreditation may address areas of concern emphasized by Hong Kong patients, such as involvement in care and emotional support from providers.

## Background

Patient experience is recognized as an essential indicator of health care quality, alongside clinical effectiveness and patient safety [1–3]. Regular collection of patient experience data allows for identification of strengths and weaknesses in health care delivery and drives quality improvement [4–6]. Public and private insurers in some countries and international accrediting bodies consider hospital performance on patient experience metrics in providing payment and certification [7, 8].

Accreditation is a practice of systematically assessing hospital performance against accepted quality standards [9]. Successful certification of accreditation signals to patients and other stakeholders that a minimum standard has been achieved [10]. This approach to quality improvement is predicated on the expectation that the accreditation exercise leads to improvement in clinical governance and quality of care [11, 12]. However, the impact of accreditation is difficult to evaluate and limited evidence exists supporting its effect on patient outcomes [13, 14].

Longitudinal comparisons of inpatient experience could provide valuable inter-organisational evaluations of quality improvement initiatives, such as accreditation. Patient experience, determined by the quality of information, communication and organisation within the healthcare setting could serve as a proxy metric for the impact of accreditation, as it informs the structure, processes and outcomes of care [15, 16]. However, little and conflicting empirical evidence is currently available to support the assumption that hospital accreditation leads to improved patient experience. One review of the accreditation literature found no significant relationship between accreditation and patient satisfaction among 20 included studies, and identified only two studies seeking to evaluate aspects of patient experience before and after accreditation with limited effect [17].

The Hong Kong public hospital system recently launched a pilot programme to test an infrastructure for accreditation of both private and public hospitals with the Australian Council on Healthcare Standards (ACHS) [18]. This study seeks to assess the longitudinal impact of hospital accreditation on patient experience through three cross-sectional evaluations conducted at a hospital participating in the pilot accreditation programme in Hong Kong.

## Methods

This study was part of a prospective mixed methods evaluation of the impact of accreditation on hospital quality, patient experience and organisational culture conducted in a large, publicly funded, university teaching hospital in Hong Kong. The hospital’s accreditation process began with a gap analysis based on ACHS standards and subsequent quality improvement initiatives to address identified gaps as described in detail elsewhere [18]. The hospital’s quality improvement activities included efforts to address hospital-wide issues, such as improving coordination, reporting and integration as well as specific department and procedure-level gaps. The current paper presents findings from the patient experience survey evaluating the effect of accreditation on care experience.

### Study subjects

Acute care inpatients aged 18 to 80 were recruited to participate in the survey on the second day of their hospital admission at three time points corresponding with accreditation: 9 months pre-accreditation (T1) as baseline, three (T2) and 15 (T3) months post-accreditation. Hospital admission and discharge staff identified eligible patients from the daily hospital admission records and prepared rosters for the research team. Patients were excluded if they were admitted to the intensive care unit, Accident and Emergency observation, isolation, labour, private, psychiatric or custodial wards, had a psychiatric diagnosis, were in poor physical status, or were unable to communicate in Cantonese, Mandarin or English. Ward nursing staff confirmed patient eligibility.

### Survey instrument

The widely used and internationally validated Picker Patient Experience Questionnaire-15 (PPE-15) was selected to evaluate participants’ experience with their recent inpatient episode. The PPE-15 measures seven aspects of inpatient experience: information and education, coordination of care, physical comfort, emotional support, respect for patient preferences, involvement of family and friends and community and transition [19]. The PPE-15 was used for the first examination of self-reported inpatient experience in Hong Kong, conducted as part of the 2005 Thematic Household Survey [3]. The 2005 data is used as a reference for this study.

Seven additional items collected patient demographic information, including education level, marital status, smoking and drinking history, self-perceived health, private health insurance coverage and medical benefits. The questionnaire was administered in Cantonese, English or Mandarin based on patient preference. Survey items were translated and back-translated by research staff and pilot tested to ensure accuracy and comprehension.

### Procedure

The same procedure was followed for each of the three data collection periods in January–March 2010, 2011, and 2012. Research staff approached eligible patients in the ward and invited them to participate. Upon agreement, research staff obtained patient consent, contact telephone numbers and preferred time for follow up phone call. Trained research staff then contacted recruited patients one-week post discharge via telephone and if unable to reach, conducted up to five additional attempts at various times of day to increase the response rate. Response rate was calculated as the number of survey respondents over the number of patients who consented to participate and were contacted by telephone.

A unique study identification number (USI) was generated for recruited patients and linked to their corresponding Hospital Number (HN). HN is a per case (admission) hospital identification number. For each recruited patient, the Admission and Discharge office provided admission ward, discharge diagnoses (ICD-9-CM; up to 15), total number of hospital admissions in the preceding year, and length of stay information. All data analysis used de-identified data. All identifying information was excluded prior to analysis by employing USI numbers as the sole form of identification in the dataset. To ensure there was no risk of personal data being identified by name, all relevant information was encrypted and stored in a separate file, with the master linking file kept in a data safe.

### Scoring and analysis

**Fig. 1:**
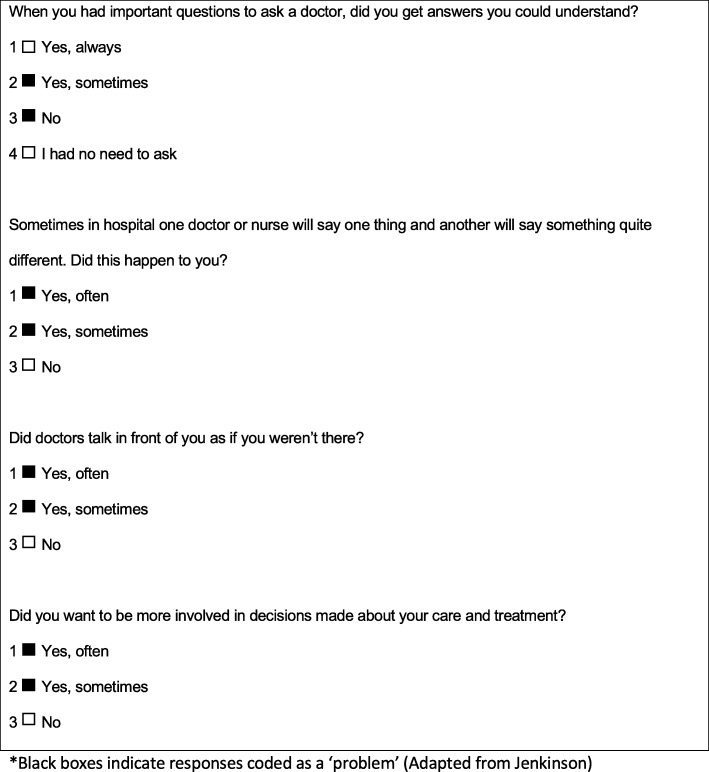
PPE-15 Sample Questions and Dichotomous Scoring

Based on the previously validated PPE scoring scheme, we coded each item dichotomously to indicate the presence or absence of a problem (See Fig. 1 for sample questions and scoring). “Problems” are aspects of the health care experience patients indicated could be improved. Summary scores were calculated as the ‘number of items identifying a problem’ over the ‘number of items answered by the patient’ on a scale of zero to 100, with zero indicating no problem and 100 indicating many problems. PPE-15 summary, domain and item scores were calculated for each collection period (T1, T2 and T3).

We first compared baseline (T1) PPE-15 scores for the study hospital to the Hong Kong public hospital scores obtained through the 2005 Thematic Household Survey (THS) [3]. The THS is a series of regular, repeated cross-sectional household surveys sampling the entire land-based Hong Kong population and covering a wide spectrum of social issues. Each survey usually focuses on two substantive policy subjects. The 2005 THS was the first to include patient experience as one of the survey sections, specifically inquiring about the most recent hospital admission in the 12 months prior to the survey. Hence, the survey items were only relevant for respondents with a recent admission and the recall period was for the entire year rather than 1 week as in our study data. We used t-tests to compare our hospital baseline scores to the 2005 Hong Kong average for public hospitals.

We then used ANOVA to evaluate differences in PPE scores across the three cross-sections with the Bonferroni post-hoc test for pairwise comparisons. Finally, we used multiple linear regression analysis with time period as the predictor variable and patient experience scores as the outcome variables to examine the effect of accreditation over time. Covariates included patient age, gender, self-reported education level, marital status, self-reported health status, smoking and alcohol use, insurance status, medical benefit status, length of stay, prior admissions and number of comorbidities. We also conducted sensitivity analyses for patients with lengths of stay (> 4 days) or number of prior admissions (> 1 prior admission) above the median since the ranges varied widely. All data analyses were conducted in STATA 13. A priori significance level of 0.05 was used for all statistical tests.

The study was approved by the Institutional Review Board of the institution and study hospital involved.

## Results

**Fig. 2:**
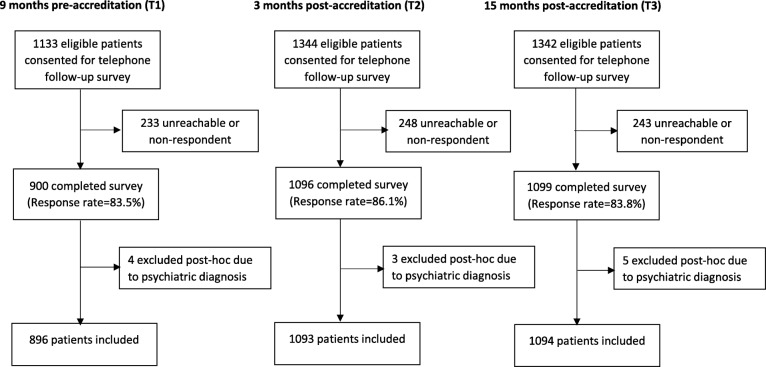
Patient Recruitment Flow Chart

Hospital staff identified 7114 patients (T1 = 2770, T2 = 2082, T3 = 2262) from the admission records meeting study eligibility criteria. Ward nurses further excluded 2556 patients they were unable to locate (T1 = 722, T2 = 291, T3 = 358), or who were unable to communicate or in too poor physical status (T1 = 526, T2 = 286, T3 = 373). Finally, 739 patients declined to participate (T1 = 389, T2 = 161, T3 = 189), leaving 3819 (T1 = 1133, T2 = 1344, T3 = 1342) patients who gave consent for telephone follow-up (See Fig. 2). Following discharge, 3083 patients (T1 = 896, T2 = 1093, T3 = 1094) completed the survey for a response rate of 83.5, 86.1, and 83.8%, respectively.

Participants did not differ by socio-demographic characteristics across the three time points (See Table 1). However, self-reported health characteristics varied by time point. T1 participants were less likely to have prior admissions and comorbidities than T2 and T3 participants, while T3 participants reported better perceived health than T1 and T2 participants.

**Table 1:** Descriptive Statistics

Category	T1 (*N* = 896)	T2 (*N* = 1093)	T3 (*N* = 1094)	*p*-value^1^
	N (%)	N (%)	N (%)	
Sex				0.19
Male	477 (53.24)	582 (53.25)	545 (49.82)	
Female	419 (46.76)	511 (46.75)	549 (50.18)	
Age				0.50
18–24	45 (5.02)	60 (5.49)	52 (4.75)	
25–34	76 (8.48)	95 (8.69)	76 (6.95)	
35–44	131 (14.62)	128 (11.71)	140 (12.80)	
45–54	173 (19.31)	211 (19.30)	234 (21.39)	
55–64	224 (25.00)	278 (25.43)	263 (24.04)	
65–80	247 (27.57)	321 (29.37)	329 (30.07)	
Median	56	56	56	0.78
Education Level				0.86
Primary or below	283 (31.62)	349 (32.02)	333 (30.75)	
Secondary	422 (47.15)	521 (47.80)	535 (49.4)	
Tertiary	190 (21.23)	220 (20.18)	215 (19.85)	
Marital Status				0.86
Never married	182 (20.31)	236 (21.71)	215 (19.93)	
Married	602 (67.19)	721 (66.33)	726 (67.28)	
Widowed/Separated	112 (12.50)	130 (11.96)	138 (12.79)	
Self-Perceived Health				0.00
Good/Very good/ Excellent	283 (31.76)	334 (30.64)	439 (40.72)	
Fair / poor	608 (68.24)	756 (69.36)	639 (59.28)	
Length of stay				
Median (Range)	4 (2–46)	4 (2–52)	4 (2–60)	0.05
Prior admission in last 12 months				0.00
0	357 (39.84)	328 (30.01)	319 (29.16)	
1	174 (19.42)	231 (21.13)	239 (21.85)	
≥ 2	365 (40.74)	534 (48.86)	536 (48.99)	
Median (Range)	1 (0–139)	1 (0–156)	1 (0–157)	0.00
Number of comorbidities				0.00
0	297 (33.15)	345 (31.56)	309 (28.24)	
1	276 (30.80)	256 (23.42)	287 (26.23)	
≥ 2	323 (36.05)	492 (45.01)	498 (45.52)	
Median (Range)	1 (0–11)	1 (0–14)	1 (0–13)	0.00

^1^Chi-square test was used to evaluate differences in groups by time point

### Patient experience scores

The hospital baseline PPE-15 scores were all significantly different from the Hong Kong public hospital average (*p* < .05) (Table 2). Hospital baseline scores for the ‘information and education,’ ‘physical comfort,’ and ‘continuity and transition’ domains were higher (worse), while ‘coordination of care’, ‘emotional support,’ ‘respect for patient preferences,’ and ‘involvement of family and friends’ domains were significantly lower (better) than the Hong Kong public hospital average.

**Table 2:** Hong Kong Public Hospital 2005 Average and Study Hospital PPE-15 Summary, Domain and Item Scores

PPE-15 Items	2005 Hong Kong Public Hospital ^1^ (*N* = 2901)	T1 (N = 896)	T2 (N = 1093)	T3 (N = 1094)	*p*-value ^2^
	Weighted% (95%CI)	Mean (95%CI)	Mean (95%CI)	Mean (95%CI)	
Information and education	**37.6 (35.0–40.2)**	**40.0 (37.3–42.6)**	**28.0 (25.8–30.3)**	**28.3 (26.0–30.6)**	**0.000**
Nurses’ answer to questions not clear	39.2 (36.4–42.1)	41.4 (38.2–44.6)	25.9 (23.3–28.5)	29.0 (26.3–31.7)	0.000
Doctors’ answers to questions not clear	34.8 (32.0–37.7)	38.5 (35.3–41.7)	30.2 (27.5–32.9)	27.6 (25.0–30.3)	0.000
Coordination of care					
Staff gave conflicting information	**30.8 (28.0–33.7)**	**30.3 (27.3–33.3)**	**24.3 (21.8–26.9)**	**17.7 (15.5–20.0)**	**0.000**
Physical comfort					
Staff did not do enough to control pain	**22.7 (20.2–25.3)**	**37.5 (33.7–41.3)**	**30.6 (26.8–34.3)**	**29.0 (25.4–32.6)**	**0.003**
Emotional support	**51.6 (49.3–53.9)**	**46.7 (44.2–49.2)**	**36.7 (34.5–38.8)**	**32.1 (30.0–34.1)**	**0.000**
Nurses did not discuss anxiety or fears	53.9 (50.9–56.9)	49.3 (46.0–52.6)	37.8 (34.9–40.7)	36.9 (34.0–39.8)	0.000
Doctor did not discuss anxiety or fears	49.6 (46.6–52.6)	41.1 (37.8–44.3)	28.4 (25.7–31.1)	30.1 (27.4–32.8)	0.000
Difficult to find someone to talk to about concerns	51.3 (48.3–54.3)	49.7 (46.6–52.9)	43.6 (40.7–46.6)	29.0 (26.3–31.7)	0.000
Respect for patient preferences	**51.9 (50.3–53.5)**	**38.9 (37.0–40.8)**	**31.3 (29.6–32.8)**	**20.5 (19.0–22.1)**	**0.000**
Not sufficiently involved in decisions about treatment and care	89.0 (86.8–91.0)	65.5 (62.4–68.6)	55.0 (52.0–57.9)	32.6 (29.8–35.4)	0.000
Not always treated with respect and dignity	37.7 (34.8–40.6)	32.2 (29.1–35.2)	25.4 (22.8–28.0)	20.9 (18.5–23.3)	0.000
Doctors sometimes talked as if I was not there	30.0 (27.3–32.8)	19.0 (16.4–21.6)	13.5 (11.4–15.5)	7.8 (6.2–9.4)	0.000
Involvement of family and friends	**48.6 (46.1–51.2)**	**46.6 (44.0–49.3)**	**35.2 (32.9–37.4)**	**34.3 (32.3–36.4)**	**0.000**
Family did not get opportunity to talk to doctor	50.7 (47.7–53.6)	39.7 (36.5–42.9)	23.6 (21.1–26.1)	21.7 (19.2–24.1)	0.000
Family not given information needed to help recovery	46.4 (43.5–49.4)	53.4 (50.1–56.7)	46.6 (43.6–49.5)	47.5 (44.5–50.5)	0.005
Continuity and transition	**28.8 (26.6–31.1)**	**43.3 (41.0–45.5)**	**42.2 (40.2–44.2)**	**36.3 (34.4–38.1)**	**0.000**
Not told about danger signals to look for at home	30.0 (27.3–32.8)	56.5 (53.4–59.7)	50.6 (47.7–53.6)	49.7 (46.7–52.7)	0.006
Purpose of medicines not explained	28.5 (25.9–31.3)	21.6 (18.9–24.3)	19.4 (17.1–21.8)	11.4 (9.5–13.3)	0.000
Not told about medication side effects	24.3 (21.7–27.0)	54.9 (51.3–58.4)	60.3 (57.2–63.4)	51.2 (47.9–54.4)	0.000
Summary Score	**41.8 (40.3–43.3)**	**41.9 (40.3–43.4)**	**34.1 (32.8–35.4)**	**29.1 (27.9–30.3)**	**0.000**

Notes: Higher scores correspond with less satisfied patients. T1 = Baseline 9 months pre-accreditation survey; T2 = 3 months post-accreditation survey; T3 = 15 months post-accreditation survey

^1^Chan SK, Wong IO, Tin KY, Fung A, Johnston JM, Leung GM. Satisfaction with inpatient care in a population-based Hong Kong Chinese sample. Quality & safety in health care. 2010;19(3):173–81

^2^ANOVA comparison of 3 time points

The boldface items indicate domains and domain scores

Overall, the PPE-15 summary scores and domain scores declined significantly (improved) over the study period (T1 to T3) (Table 2). Between T1 and T2, all domain scores declined except for the ‘continuity and transition’ domain, reflecting high scores for two items related to medication information. Between T2 and T3, the ‘continuity and transition’ domain showed significant improvement, while other domains continued to improve (i.e. ‘care coordination’, ‘emotional support’, ‘respect for patient preferences’ and ‘involvement of family and friends’) or hold steady (i.e. ‘information and education’ and ‘physical comfort’).

The multiple regression results (Table 3) confirmed the time point score comparisons. The T2 and T3 parameter estimates were declining (improving) for PPE-15 summary scores and all domain scores except for the ‘continuity and transition’ domain, for which the declining coefficient was only significant at T3. When adjusted for all covariates, the multiple regression parameter estimates were similar to the unadjusted estimates. Likewise, the sensitivity analyses confirmed similar findings among patients with multiple prior admissions and longer lengths of stay (> 4 days) (data not shown). Patients with longer stays generally evaluated their experience more favourably (lower scores) than the full sample, while patients with multiple prior admissions indicated more problems. However, trends in patient experience scores among patients with longer stays and patients with multiple prior admissions were generally consistent with the full sample except for the ‘physical comfort’ domain, which while declining was not significant at T2 or T3 for patients with longer stays and T3 for patients with multiple prior admissions.

**Table 3:** Multiple Regression with Time Point (Post-accreditation) as Predictor of PPE-15 Domain and Summary Scores

PPE-15 Domains		T1 (N = 896)	T2 (N = 1093)		T3 (N = 1094)	
			Parameter estimate (95% CI)	*p*-value	Parameter estimate (95% CI)	*p*-value
Information and education	Adjusted	Ref.	−11.9 (−15.3,-8.5)	0.000	−11.7 (− 15.1,-8.2)	0.000
		Ref.	−12.3 (− 15.7,-8.8)	0.000	− 11.2 (− 14.7,-7.8)	0.000
Coordination of care	Adjusted	Ref.	−6.0 (−9.7,-2.2)	0.002	−12.6 (− 16.3,-8.8)	0.000
		Ref.	−6.4 (− 10.2,-2.7)	0.001	−11.9 (− 15.6,-8.1)	0.000
Physical comfort	Adjusted	Ref.	−6.9 (−12.2,-16.6)	0.010	−8.5 (−13.7,-3.3)	0.001
		Ref.	−8.2 (−13.5,-2.9)	0.002	−8.8 (−14.1,-3.5)	0.001
Emotional support	Adjusted	Ref.	−10.0 (−13.3,-6.8)	0.000	−14.6 (−17.9,-11.4)	0.000
		Ref.	−10.6 (−13.8,-7.4)	0.000	−14.0 (−17.3,-10.8)	0.000
Respect for patient preferences	Adjusted	Ref.	−7.7 (−10.1,-5.2)	0.000	−18.4 (−20.8,-16.0)	0.000
		Ref.	−8.0 (−10.4,-5.7)	0.000	−18.6 (−21.0,-16.2)	0.000
Involvement of family and friends	Adjusted	Ref.	−11.5 (−14.8,-8.2)	0.000	−12.3 (−15.6,-9.0)	0.000
		Ref.	−12.3 (−15.6,-8.9)	0.000	−11.6 (14.9,-8.2)	0.000
Continuity and transition	Adjusted	Ref.	−1.1 (−4.0,1.8)	0.470	−7.0 (−9.9,-4.1)	0.000
		Ref.	−1.8 (− 4.7,1.1)	0.218	−7.3 (−10.2,-4.4)	0.000
**Summary score**	Adjusted	Ref.	−7.8 (−9.7,-5.9)	0.000	−12.8 (−14.7,-10.9)	0.000
		Ref.	−8.4 (−10.2,-6.5)	0.000	−12.6 (−14.5,-10.7)	0.000

Note: Covariates in the adjusted model include patient age, gender, self-reported education level, marital status, self-reported health status, length of stay, prior admissions and number of comorbidities, smoking habit, alcohol use, insurance and medical benefit status

## Discussion

To our knowledge, this is the first attempt to evaluate the effect of accreditation longitudinally using patient experience scores. Our findings indicate robust improvements in patient experience in the study hospital following accreditation with continued or sustained improvements at 15 months post-accreditation. These results provide initial support for the positive potential of the hospital accreditation exercise to enhance patient experience.

Prior studies evaluating the effect of accreditation on patient experience have mostly been observational comparing patient experience scores between accredited and unaccredited hospitals, with little support for accreditation improving patient experience [17, 20–22]. Given these findings, a prior narrative review of the health service accreditation literature by Hinchcliff and colleagues concluded accreditation may target aspects of health care less visible to patients [23]. However, the improving patient experience scores in our study following accreditation suggest accreditation indeed reached the patient sphere. In fact, the domain showing the greatest absolute change over the study period was “respect for patient preferences,” which declined (improved) significantly at both T2 and T3. Given the significant resource expenditures and increased staff workload required to facilitate the accreditation process and despite accreditation’s often-questioned utility, this study suggests patients may benefit from the extensive effort [9].

This study also provides new understanding of patient experience with care in Asia. Only a few studies to date have evaluated patient experience within an Asian context, identifying different priorities for care than in western contexts [24]. The 2005 THS data presented as a benchmark for our study indicate not only a higher proportion of problems relative to Western contexts, but also different types of problems [3]. Consistent with our study data, the most commonly reported problem in the 2005 THS was “not sufficiently involved in decisions about treatment and care,” indicated by 89% of THS respondents who attended a public hospital and 66% of our respondents at baseline, more than double the proportion indicating this as a problem in western contexts [3]. Likewise, lack of emotional support from doctors and nurses to discuss fears and anxieties was a key complaint in both the THS and study hospital data, differing from emotional support concerns in western contexts where patients are more likely to indicate difficulty finding someone to talk to about concerns [3]. Chan and colleagues speculate that discrepancies between Hong Kong and western patients’ perceptions of care may reflect health care providers “not schooled or conditioned in the patient-centred participatory tradition of care, which per se has been a fairly recent development in the West.” [3] Findings from our study suggest the accreditation effort may especially enhance these disparate areas, improving patient perception of involvement in care and emotional support from providers.

Although our study provides compelling evidence of positive effects of accreditation on patient experience, it is not without limitations. First, this study was exploratory research aiming to assess the impact of hospital accreditation on patient experience in one hospital. While we would have preferred to compare multiple hospitals with and without accreditation interventions, only the single case study was feasible and thus results may not be generalizable to other hospitals. Second, given hospital patient experience surveys evaluate patient care episodes, we were not able to collect data from the same patients for multiple time points, and thus our data represents three cross-sections. Although we sought to ensure data from the three cross-sections were comparable through identical recruitment procedures, T3 subjects were more likely to report better self-perceived health and comorbidities, which may have influenced raw patient experience scores. However, our adjusted analyses controlling for these covariates confirmed the unadjusted results. We also sought to minimize both recall and response fear bias by recruiting during the inpatient stay but conducting the survey 1 week post-discharge.

## Conclusion

Despite limited evidence supporting its effect on patient outcomes, accreditation continues to thrive as an international industry, and essential and often compulsory quality improvement activity [9–11, 14]. This study provides initial evidence of the positive potential of the hospital accreditation exercise to enhance patient experience. Moreover, it suggests the quality improvement initiatives associated with accreditation may address areas of concern emphasized by Hong Kong patients, such as involvement in care and emotional support from providers. Further studies should assess the effect of accreditation on patient experience in different settings including both accreditation intervention and non-intervention sites. Moreover, additional studies should investigate the particular interventions related to accreditation that may have influenced patient experience. As hospitals continue to pursue accreditation going forward in spite of limited evidence of other positive effects, they should seek to harness its potential to improve patient experience.

## Data Availability

The (de-identified) datasets generated and analysed during the current study are available from the corresponding author on reasonable request.

## Abbreviations

ACHS: Australian Council on Healthcare Standards
HN: Hospital Number
ICD-9-CM: International Classification of Diseases, Ninth Revision, Clinical Modification
PPE-15: Picker Patient Experience Questionnaire-15
T1: Time 1 (baseline, 9 months pre-accreditation)
T2: Time 2 (three months post-accreditation)
T3: Time 3 (fifteen months post-accreditation)
THS: Thematic Household Survey
USI: Unique study identification number
